# Implementation of motivational interviewing in the general practice setting: a qualitative study

**DOI:** 10.1186/s12875-022-01623-z

**Published:** 2022-01-28

**Authors:** Saskia M Boom, Riëtta Oberink, Abigail J E Zonneveld, Nynke van Dijk, Mechteld R M Visser

**Affiliations:** Department of General Practice/ Family Medicine, Amsterdam University Medical Centers, Meibergdreef 15, 1105 AZ Amsterdam, Netherlands

**Keywords:** Motivational interviewing, Implementation, Family medicine, General practitioner, Practice Nurse

## Abstract

**Background:**

General Practitioners (GPs) and Practice Nurses (PNs) collaboratively play an important role in preventing and monitoring chronic diseases. They are trained in Motivational Interviewing (MI), which is a communication style to intrinsically motivate patients to a healthier lifestyle. However, being trained in MI skills does not necessarily mean that it is implemented in daily practice so patients actually benefit. The aim of this study is to identify factors that facilitate or impede the implementation of MI in General Practice.

**Methods:**

A total of 152 participants (93 GP-trainees and 59 PN-trainees) who were trained in MI completed a questionnaire regarding the implementation of MI. Semi-structured interviews (*N* = 17) were conducted with GPs and PNs (ranging from almost graduated to highly experienced) who were selected through the process of maximum variation sampling. The interview guide was based on the five-stage implementation model of Grol and Wensing.

**Results:**

Thirteen factors that influence the implementation of MI in General Practice were identified. They can be allocated to three categories: (1) setting factors such as time, (2) GP/PN factors such as self-efficacy, and (3) patient factors such as cultural background.

**Conclusions:**

Overall, GPs and PNs considered MI to be useful and part of their professional responsibility. Most difficulties become apparent in stage 4 (change: applying MI skills in practice) and 5 (consolidation: integrating MI into daily routine and embedment in organisation) of Grol and Wensing’s model. Therefore, it is important that training does not only focus on MI skills. It is essential to pay explicit attention to the factors that impact implementation, as well as the appropriate tools to tackle the barriers. These insights can help trainers to effectively support GPs and PNs to apply and maintain their MI skills in daily practice.

**Supplementary Information:**

The online version contains supplementary material available at 10.1186/s12875-022-01623-z.

## Background

The goal of training healthcare practitioners is to contribute to their knowledge, skills, and attitude to ultimately improve the health of their patients [1]. However, do these training efforts transfer to daily practice to such an extent that patients actually benefit [2–4] ? The loss of translation between the acquired competencies during training and application of these skills in routine practice has been described as the “Achilles heel” of the training process [5]. Therefore, it is important not to solely focus on training healthcare professionals in evidence-based practices, but also to help them apply these skills in daily practice by exploring factors that affect implementation.

In this study, we address the implementation of Motivational Interviewing (MI) in the General Practice setting. MI invites people into collaborative conversations to increase intrinsic motivation towards behaviour change [6]. The importance of supporting patients to adopt a healthier lifestyle has gained recognition, and the initial focus on curative healthcare has shifted towards incorporating prevention and monitoring of chronic diseases [7, 8]. In the Dutch General Practice setting, General Practitioners (GPs) and Practice Nurses (PNs) collaboratively take on this role and, therefore, MI training is a compulsory component of their training program [9, 10]. However, being trained in MI skills does not necessarily mean that MI is applied in routine practice [11, 12] Various studies have been conducted to address the implementation of MI in healthcare settings [e.g. [13–16]]. Some of these studies indicate potential barriers, but these are mainly expressed in quantitative data, or only briefly mentioned without elaboration. Additionally, as implementation is context-specific, studies that focus on other settings or professions (e.g. dieticians), or target one specific health issue are less informative for the General Practice setting with a multitude of different health-related problems [17]. Additionally, in contrast to previous studies, we incorporate both professions in the General Practice setting who are trained in MI, and we use a theoretical framework to address all five stages that are needed for successful implementation according to Grol and Wensing [18]. The aim of this study is to identify the factors that affect the implementation of MI in the General Practice setting.

## Methods

### Setting

In the Netherlands, GPs complete a three-year post-graduate GP Specialty-Training. For PNs, a two-year training program is required after achieving secondary vocational education or higher professional education. MI training is incorporated in the curricula of both GP-trainees and PN-trainees. They combine their educational program (one day a week) with work under supervision in a General Practice setting, which provides the opportunity to practice MI skills.

### Design

Qualitative data is based on questionnaires and semi-structured interviews. A constructivist perspective was adopted, which involves the assumption that there is no absolute truth that can be discovered, but it attempts to understand the perspectives and experiences of the participants.

### Participants

#### Questionnaire participants

We invited all GP-trainees at the Amsterdam UMC, location AMC, and PN-trainees of seven different educational programs who started their MI training between November 2015 and December 2016 to participate in our larger research project on the assessment, training, and implementation of MI. A total of 107 GP- and 91 PN-trainees agreed to participate. For this study, the implementation questionnaire and background information of these participants was used.

#### Interview participants

Interviewees were selected through the process of maximum variation sampling with the aim of generating a heterogeneous sample. They differed regarding gender, age, profession, experience (in patient care and MI), and time passed since they completed their last MI training.

Ten interviewees were selected from our larger research project and, as a result, additional information was available. This allowed for a more precise process of maximum variation sampling, based on variation in intention to continue to apply MI, and MI skills as measured by the VASE-(M)HC. This video-based instrument is based on the VASE-R [19], and adjusted and validated for the General Practice setting [20]. The other interviewees were recruited via the network of colleagues to further diversify the sample.

#### Ethical considerations

All participants received written information about the study, had the opportunity to ask questions, and signed informed consent. No incentives were offered to the participants who completed the questionnaires. The interviewees received a gift voucher of 20 euros. We obtained ethical approval from the Ethical Review Board of the NVMO (Dutch Association for Medical Education, NERB 422); all methods were performed in accordance with the relevant guidelines and regulations.

### Data collection

#### Questionnaire

The implementation questionnaire consisted of open-ended questions to gain insight into their experience with the application of MI in daily practice and to identify factors that facilitate or hinder the implementation.

#### Interviews

The model of Grol and Wensing [18] was used as a framework to design the interview guide. This five-stage model (Table 1) provides structure for the implementation of innovations in healthcare settings and helps to identify which of these stages are affected by potential barriers that should be addressed to improve implementation. In order to provide a more comprehensive picture of what each stage entails, examples of interview questions are presented in Appendix A. The first author (SB) conducted a pilot interview with a former participant of her MI training, to ensure the questions were clear and suited to the study objective. No revisions were made to the interview guide. However, the importance of differentiating between the actual experience of the interviewees themselves instead of what had heard from their trainer became especially apparent. The following interviews were conducted by SB (*N* = 1), and two research interns; AZ (*N* = 10) and LF (*N* = 6). Interviews lasted 26–82 (average 55) minutes and were transcribed verbatim.

**Table 1:** The five-stage implementation model of Grol and Wensing (18)

Stage	Description, adjusted to MI and the General Practice setting
(1) Orientation	GPs/PNs hear about MI and they become interested in learning more about it
(2) Insight	GPs/PNs gain an understanding of what MI entails, how it can affect their way of working and they get prepared by learning MI (e.g. attending a training)
(3) Acceptance	GPs/PNs develop a positive attitude towards MI. They consider MI to be useful and feasible, and they have the intention to apply the acquired MI skills in practice
(4) Change	GPs/PNs start to apply MI in their daily practice and experience its value
(5) Consolidating change	GPs/PNs integrate MI into their daily work and skills are consolidated. MI is embedded within their organisation

### Analysis

The answers to the open-ended questions of the questionnaire and the interviews were coded using MAXqda [21]. The qualitative data was examined through thematic analyses [22]. Both coders started with a general overview of the data, as AZ had conducted ten interviews and SB read all 17 transcripts. They both open coded one interview independently. Codes were compared and the resulting coding scheme was used to code the same set of three additional interviews independently. Coding differences were discussed until consensus was reached. If relevant, the coding scheme was adjusted accordingly and discussed with the research team.

Subsequently, AZ coded four and SB coded another nine interviews as well as the open-ended questions of the questionnaire. Uncertainties were discussed. After 17 interviews thematic saturation was reached [23]. Finally, SB and a third research intern (MN) re-examined all interviews to ensure that the themes and quotes as depicted in the results-section provide a good representation of the data. Standards for Reporting Qualitative Research (SRQR) [24] were followed to design and report the qualitative data.

## Results

### Characteristics of participants

GP-trainees (*N* = 93) and PN-trainees (*N* = 59) completed the questionnaire. Eight GPs and nine PNs, ranging from almost graduated to 43 years of experience in patient care, were interviewed. Participant characteristics are presented in Table 2.

**Table 2:** Characteristics of the participants

	**Questionnaire (*N* = 152)**		**Interviews (*N* = 17)**	
	***N***		***N***	
**Age**	149		17	
Mean (SD) [Range]		34.0 (8.1) [22–58]		35.9 (9.9) [27–62]
**Self-identified gender: N (%)**	152		17	
- Male		34 (22%)		5 (29%)
- Female		118 (78%)		12 (71%)
**Education/ profession: N (%)**	152		17	
- GP		93 (61%)		8 (47%)
- PN		59 (39%)		9 (53%)
**Prior education PNs: N (%) (N/A for GPs)**	55 (of 59 PNs)		9 (of 9 PNs)	
- Secondary vocational education or lower		17 (31%)		3 (33%)
- Minimum of higher professional education		38 (69%)		6 (67%)
**Experience with patient care (years)**	143		17	
Mean (SD) [Range]		9.0 (8.3) [1–40]		9.8(10.1) [2–43]
**MI training (hours)**	141		17	
Mean (SD) [Range]		11.5 (5.7) [8–45]		15.6 (13.5) [6–56]
**Time passed since last MI training: N(%)**	152		17	
- < 6 months		152 (100%)		6 (35%)
-6 months—1 year				8 (47%)
- > 1 year				3 (18%)
**MI skills based on (VASE-M)HC: scoring range 0–54**	149		10	
Mean (SD) [Range]		31.7 (7.4) [10–50]		32.8 (10.3) [13–45]

*GP*  General Practitioner, *PN* Practice Nurse, *MI* Motivational Interviewing, *SD* Standard Deviation, *N/A* Not Applicable, *VASE-(M)HC* Video Assessment of Simulated Encounters – (Mental) Health Care, a video-based instrument to assess MI skills that is adjusted and validated in the General Practice setting [20]. The number of MI training hours is based on experience both prior to and during the study. This last category is based on the training groups per institute, however, individual attendance may differ

**Table 3:** Factors affecting the implementation of MI in General Practice

1. Setting factors
- Time
- Combination with other tasks
- Continuity
- Recognizing opportunities
- Teamwork
2. GP/PN factors
- Introduction to MI
- Perception of professional responsibility
- Usefulness
- Self-efficacy
- Ingrained habits
3. Patient factors
- Level of understanding
- Age
- Culture

### Factors affecting the implementation of MI in general practice

Findings derived from the open-ended questions and interviews resulted in thirteen factors, allocated to three categories; setting factors, GP/PN factors, and patient factors (Table 3). Illustrative quotes in addition to the text below are provided in Appendix B to provide an enriched picture.

### Category 1: setting factors

#### Time

Limited time was perceived to be a barrier: “*If I know I’m running late and three more patients are waiting, (…) I usually don’t apply it [MI]*” (Interview(I)-GP6). Time pressure also has indirect influences, for example, as it can affect the demeanour of the practitioner: "*then you are less likely to adopt that relaxed attitude*" (I-PN2). Nonetheless, a practitioner stressed: "*if I have the feeling ‘we are on to something’, I think it is so valuable that I briefly pay attention to it*" (I-GP3) even if this will cause him to run late.

#### Combination with other tasks

Concerns regarding the combination of MI with other tasks, were mainly related to competing demands on limited time. The patients' agenda and medical needs often take priority and *“sometimes history taking and physical examination take up all the available time”* (I-GP4). However, "*If I notice it is more beneficial to (…) solely communicate (…) or try to motivate (…) then I switch my focus to that and plan another appointment (…) for the [physical] check-ups"* (I-PN7). PN(I-4) noticed that MI helps to place other tasks in a different perspective: "*[Because] you have to tick off all the necessary healthcare measures, you sometimes lose focus regarding the patients' self-management or goals. So the focus might have shifted more towards what is important for the patient, (…) because of MI*".

#### Continuity

To overcome time restrictions, participants mentioned "*it helps to make a follow-up appointment*” (I-GP5). However, this is often not possible for GPs in a locum position; “*If I am unable to see the patient again, then I won’t use it. Yes, at most a certain technique, but I’m not going to map it out completely*” (I-GP2). Therefore, *“it’s easier to apply it to your own patients […] you see them frequently, so you can make long-term plans”* (I-GP4). Also, *"if I know a patient very well (…) you feel responsible (…) due to that bond you’ve formed, and then I am more likely to apply MI, despite time restrictions"* (I-GP6).

#### Recognizing opportunities

GP(I-5) mentioned: “*During the training, I became aware that you can apply it [MI] in many more situations than I first anticipated*”. However, a PN(I-4) emphasized that recognizing all these opportunities in the General Practice setting is more challenging compared to his previous job at an STD (Sexually Transmitted Disease) clinic, where he only had to focus on one type of health behaviour. Multiple participants mentioned it is especially difficult “*to think about it [MI] during less obvious scenarios*” (Questionnaire(Q)-GP161), for example, when "*elderly do not want to use a hearing aid, which is dangerous if they go outside in traffic*" (I-PN4).

#### Teamwork

Participants stated it is preferable if "*coworkers (including the older generation) would apply it [MI] as well"* (Q-GP173). It is helpful to "*have someone at work to motivate each other to continue to engage with MI (…) and to keep reflecting on it* " (I-PN4). However, one PN(I-2) used the different approach of the GP as an advantage: "*If it really is time to take action, then I sometimes ask the doctor to join our conversation, to be the bad cop and to make a statement, so that I—as a good cop—can use that to build on that*".

### Category 2: GP/ PN factors

#### Introduction to MI

For some interviewees, MI was a compulsory course in their specialty-training. “*I remember some GP-trainees wondered to what extent a GP should master those skills (…) Personally, I thought it was beneficial that it was presented as something that is part of the job. That’s why I chose to quickly master it [MI]. (…) And then I noticed the results, which was especially eye-opening*” (I-GP2). Interviewees who graduated before MI became part of the curriculum, developed interest as “*it was the ‘new thing in town’, it was everywhere*” (I-GP3).

#### Perception of professional responsibility

Participants from both professions considered the use of MI to address health behaviour as an important part of their work. "*As a GP you often have the opportunity to (…) raise the patient’s awareness how he can contribute to his own health (…) and to empower him (…) it is a huge missed opportunity if you don’t do that*" (I-GP3). However, some emphasised that it is "*especially important for PNs to apply MI as they have more time [than GPs] (…) and they focus on lifestyle*" (I-GP1).

#### Usefulness

A few participants thought the results concerning actual improved lifestyle were occasionally disappointing; “*when I notice that it doesn’t seem to work with a patient, I discontinue*"(I-GP2) and if “*ten colleagues have tried it already, why would I succeed?*” (I-GP6).

Nonetheless, most experienced that MI benefits patients in many ways, even if a patient did not change his behaviour; “*they feel heard and taken seriously*” (I-GP7), and “*it makes them more aware of their own responsibility regarding their health*” (I-PN9).

Participants voiced that MI also increases their job satisfaction. They get to know their patients and the unique reasoning behind their behaviour. “*As every conversation is different, it brings about a lot of variation*” (I-PN2). Aspects such as “*experiencing that it works, feeling less dissatisfied*” (Q-GP135) acted as stimulants to apply MI. Some mentioned that despite the initial time investment they eventually even gained time by using MI.

#### Self-efficacy

A few practitioners indicated that "*insufficient confidence in their own ability*" (Q-GP143) acted as a barrier, for example, because "*I still have to think very carefully when applying MI which causes the conversation to get stuck*" (Q-PN234). However, it was mostly emphasised that "*undoubtedly, it’s not perfect, but everything you mention could make someone think differently, it could still help*” (I-GP5).

A PN voiced that MI is especially "*demanding for PNs, because of their [educational level], while MI requires academic insight. Certain elements can be done by a PN, but [MI] calls for a helicopter view that is related to insight, intelligence, flexibility to abandon the protocol*" (I-PN3). Additionally, it was mentioned that it can be overwhelming for novice practitioners as there are so many other topics and skills to learn. As they gained more experience in general, they also felt more comfortable to use MI.

#### Ingrained habits

Participants recognized the risk of falling back into ingrained routines. For example, moving so fast that “*you're already on the train, while the patient is still on the platform*” (I-PN3) due to time pressure, or “*prematurely assuming that somebody doesn’t want to change*” (I-GP4) when being low on energy. GP(I-1) mentioned it is good to learn MI at an early stage, because “*then you’re still a blank slate (…) and there are less ingrained habits that easily resurface*”.

### Category 3: Patient factors

Interviewees addressed that MI might not work with every patient: "*one size doesn't fit all*" (I-PN1) and with "*some patients you just need to be firm and tell them 'quit smoking now' (…) whilst with others, you absolutely shouldn't do that*" (I-PN7). Even though every patient is unique, certain group characteristics were mentioned to affect the perceived degree of fit with MI.

#### Level of understanding

GP(I-4) emphasised that lifestyle changes often involve long-term effects that are more challenging to discuss with "*people who are less intelligent, [as they] are less likely to think ahead. (…) It's hard to convey that high blood pressure is not that important right now, but it will be in 10 years."* Additionally, they seem to *"have less self-reflection and self-understanding*" (I-PN4). A PN commented "*it is more difficult to apply, but I try. Sometimes it works, but often after one question they are like: "It’s enough for today*" (I-PN8).

Additionally, practical barriers were mentioned; "*social deprivation is especially difficult because those people actually have fewer opportunities in reality*" (I-GP3). One GP raised the influence on the practitioner: "*you also have less hope (…) then it’s more focused on merely 'patching up' the urgent problems*" (I-GP3). Interviewees noted the intelligence level of patients is often not listed in the Electronic Health Record (EHR) and pointed out the risk of overestimation: "*Sometimes you can be so mistaken regarding the intelligence of people (…) You constantly have to be aware of that"* (I-PN1).

#### Age

Participants stated "*when they are very young they are less capable to tell why they do or do not do something (…) They have to be a little older, around 10–12, so they are able to express themselves well and take more responsibility for, let’s say, their asthma medication*” (I-GP5). Others mentioned that MI is more difficult to apply with relatively older patients, above the “*age of 60–70* (…) *they seem to be more set in their ways"* (I-PN6).

#### Culture

Some interviewees thought that MI should be possible with patients from different cultural backgrounds. Others stated that it is more difficult as culture affects mutual understanding. This cannot simply be solved by inviting a family member or interpreter because "*I think that language is more than just a word that has been translated*" (I-PN1).

Even if language is not a problem, cultural differences can act as barriers. Interviewees thought it to be more difficult to place themselves in the patient’s situation, and struggle with differences in communication style, expectations and health beliefs; "*they are used to a different way of communicating with the doctor, they say 'yes' to everything and they are less used to think along and play an active role (…) I have the impression that they find that type of [MI] questions more difficult* " (I-GP5). Another example was described by a GP: "*There are certain cultures, such as the African culture (…) If they don't feel their high blood pressure (…), they do not take blood pressure medication. As soon as they feel weak, they take a few pills (…), but as soon as they feel better, they don't take them anymore. (…) It is difficult to motivate [patients with chronic disease] to eat less salt, to lose weight, if they don’t experience symptoms on a daily basis (…) I really think that is cultural. (…) A doctor who works in [local area] has many African patients and he said their perception of illness is different*" (I-GP4).

## Discussion

### Summary

Analysis of questionnaires and interviews with GPs and PNs identified thirteen factors, allocated to three categories; (1) setting factors, (2) GP/PN factors, and (3) patient factors. Placing these factors into the Grol and Wensing implementation model [18], it appears that stages 1 (orientation) and 3 (acceptance) are successfully run through by the participants. Addressing health behaviour of patients is perceived as an important task of GPs and PNs. They noticed benefits of MI for the patients as well as a positive contribution to their own job satisfaction. However, several factors hinder the application of MI affecting stage 4 (change) and 5 (consolidating change). The emphasis of MI literature, research, and training is often largely on MI skills. However, implementation can be improved by explicitly addressing perceived barriers and how to overcome them in stage 2 (insight) and additional coaching during stages 4 and 5.

It could be helpful to incorporate training videos to illustrate the possibilities of MI in more challenging conditions, such as consultations with patients with a limited level of understanding or a different cultural background, and to demonstrate what is helpful in those specific situations. Trainees could be encouraged to practice this with actors or with their own patients, while receiving feedback and additional tools.

Also, it could be useful to consider the incorporation of innovations that can help to overcome the implementation barriers. E-health self-management support programs that focus on lifestyle and behavioural changes cannot replace MI consultations, but they can be of added value [25]. For example, it can empower patients to take charge of their own health, which is congruent with the spirit of MI, and they are not dependent on the amount of time that the practitioner has available. Moreover, because the assignments encourage patients to think about their values and other aspects that promote change, the consultation time can be used to elaborate on this in more in-depth conversations and for other aspects that require face to face interaction.

### Comparison with existing literature

Positive changes regarding the implementation of MI in General Practice are already noticeable. Interviews conducted in 2009 indicated that there was a resistance against preventative tasks among Dutch GPs and PNs [26]). They recognized the importance of motivation in order to quit smoking successfully, but did not consider motivating patients as their responsibility. PNs mainly favoured medications, while GPs often used confrontational methods and were unaware of other counselling styles. The current study demonstrates that GPs and PNs consider it important to motivate their patients, and experience MI as a useful way to do so. On the other hand, this study also reveals that there are still important steps to take regarding the application and consolidation of MI in daily practice. It is especially noteworthy that there seems to be a gap in the literature between studies that describe the possibilities of MI and the actual experience of practitioners. For example, MI is suggested to be efficacious even in brief consultations and with patients across different ages and ethnicities [27–30]. However, the fact that research indicates that this is possible, does not mean that it is also feasible in daily practice. Interview studies echo our finding that practitioners experience aspects such as time pressure and certain patient characteristics (e.g. level of understanding and different cultural background) as important obstacles to effectively address health behaviour [31–35]. Moreover, consistent with the notion that experiences influence the energy and motivation for  practising MI [33], practitioners in our study espoused that their perceived lack of effect in certain interactions discouraged them to apply MI in similar situations. As these experiences may reduce the use of MI, we consider it highly important to take the perceived barriers seriously.

### Strengths and limitations

Strengths of this study are the combination of questionnaires and in-depth interviews, the inclusion of both professions (GPs and PNs) who use MI in the General Practice setting, the heterogeneity of the participants and distinct professional backgrounds of the researchers to ensure that the data is interpreted through a lens of different experiences and perspectives. We also used a theoretical framework, to address all the stages and aspects that are considered important for successful implementation.

It is noteworthy that most participants consider MI to be useful and part of their professional responsibility. This might be influenced by two potential limitations. Firstly, practitioners willing to partake in the interviews may be particularly interested in MI. To minimise this bias risk, we included GPs and PNs who completed the training a long time ago, as initial positive expectations might decline over time, as well as practitioners who did not voluntarily chose to attend an MI training [4, 36]. Additionally, we invited GPs and PNs from our larger research project with relatively poor MI skills or low intention to keep using MI, as these might be relevant indicators of experienced implementation barriers. However, only 4% of the respondents scored somewhat lower (3 or 4 on a scale from 0–7) regarding their intention to keep using MI.

Secondly, a common source of bias is social-desirable answers [37]. However, similar findings were reported in the questionnaires that were conducted anonymously. Additionally, even though the majority of the participants considered it important to apply MI, they were not solely positive about the implementation in daily practice. This indicates that they felt they could talk candidly about the more difficult aspects they encountered. Another potential limitation is the apparent strong gender and age bias in those who participated in the study. In regards to gender, this is in line with the current trend of an increasing percentage of female GPs (from 33% in 2005 to 58% in 2019) [38]. Amongst PNs the percentage of females is even higher (96%) [39]. The average age of our participants is relatively young compared to the Dutch GPs and PNs who currently work in the general practice setting. However, to gain insight into the implementation of MI, we only included practitioners who completed an MI training, which mainly applies to the younger generation.

## Supplementary Information

**Additional file 1.** The five-stage implementation model of Grol and Wensing and examples of accompanying questions.

**Additional file 2.** Additional quotes related to factors influencing implementation of MI in General Practice.

## Data Availability

The data, questionnaires and transcripts, analysed during the current study are available from the corresponding author on reasonable request.

## Abbreviations

MI: Motivational Interviewing
GP: General Practitioner
PN: Practice Nurse
