# Supplementary material for: Implementation of motivational interviewing in the general practice setting: a qualitative study
Source: BMC Prim Care. 2022 Jan 28;23:21. doi: 10.1186/s12875-022-01623-z (PMC8800318; doi:10.1186/s12875-022-01623-z)
Supplement: Supplementary file 1 — Additional file 1. The five-stage implementation model of Grol and Wensing and examples of accompanying questions. [file 12875_2022_1623_MOESM1_ESM.docx]

**Appendix A.
The five-stage implementation model of Grol and Wensing and examples of accompanying questions**

| **Description, adjusted to MI and the General Practice setting** | **Examples of questions of the interview guide** |
| --- | --- |
| **Stage (1) Orientation** | |
| GPs/PNs hear about MI and they become interested in learning more about it. | When and how were you first introduced to the topic of MI?  What were your initial thoughts and expectations of the use of MI in practice? |
| **Stage (2) Insight** | |
| GPs/PNs gain an understanding of what MI entails, what it would mean for their way of working, and they get prepared by learning MI (e.g. attending a training). | To what extent did you gain insight into your own MI skills during the training? How competent did you feel in applying MI?  What are the most important and valuable aspects of MI that you have learned during training? Why? |
| **Stage (3) Acceptance** |  |
| GPs/PNs develop a positive attitude towards MI. They consider MI to be useful and feasible, and they have the intention to apply the learned MI skills in practice. | To what extent do you think that MI can contribute to the improvement of health care? Usefulness? Importance? Added value?   How important is MI for you to be able to deliver good care? And for GPs and PNs in general?   To what extent did you have the intention to apply MI in practice upon completion of the training?   To what extent did you think that it is feasible for GPs and PNs to use MI in daily practice? |
| **Stage (4) Change** |  |
| GPs/PNs start to apply MI in practice and experience its value. | Did your experience of applying MI in practice influence your perception of its usefulness? And its feasibility? And your feeling of competence? If so, in what way?  In which situations were you (not) content with the application of MI? Why? What was the underlying reason(s) according to you? Can you provide case examples to illustrate this?  Do you notice differences (for the patient and/ or yourself) as a result of using MI? If so, which differences? |
| **Stage (5) Consolidating change** |  |
| GPs/PNs integrate MI into their daily work and skills are consolidated. MI is embedded in their organisation. | To what extent are you able to integrate MI in your daily practice? What makes it easier or more difficult?  What would help you to keep applying MI in your daily work?   At what moment is it likely that you fall back into old routines/ ingrained habits? How do you prevent this?  How is MI perceived by colleagues of the practice where you work? Do they apply MI themselves? Do they accept/ support it? |
