# Supplementary material for: Implementation of motivational interviewing in the general practice setting: a qualitative study
Source: BMC Prim Care. 2022 Jan 28;23:21. doi: 10.1186/s12875-022-01623-z (PMC8800318; doi:10.1186/s12875-022-01623-z)
Supplement: Supplementary file 2 — Additional file 2. Additional quotes related to factors influencing implementation of MI in General Practice. [file 12875_2022_1623_MOESM2_ESM.docx]

**Appendix B.
Additional quotes related to factors influencing implementation of MI in General Practice.**

| **(Sub)categories** | **Examples of quote** |
| --- | --- |
| **1. Setting factors** |  |
| Time | “*For me, it’s mainly my short-term thinking. That’s who I am, and I think, like most doctors do, today I have a busy day already, so it will take me too much time. And the notion that it can save me time in the future, that is less appealing to me I think.*” (I-GP6)  *"That is the problem: time. I think that is the big pitfall (...) you need to have enough time, otherwise it is pointless." (I-GP1)* |
| Combination with other tasks | Questionnaire-What makes it difficult for you to apply MI in daily practice?  “*Often 10 minutes are already "lost" with evaluating how things are going, what changes have taken place and the question that the patient wants to discuss today. Only then MI starts and within 10 minutes the next patient is already waiting. Then everything must be discussed, summarized, a follow-up appointment must be planned and everything must be entered [in the computer].”* (Q-PN106) |
| Continuity | “*If I work there for only one day, I feel less responsible.*” (I-GP6)  "*You can start [with MI], but if you cannot follow up, because you are there for a short time in a locum position, then it is not worthwhile. (...) I think you should be able to see your patients regularly. That you work somewhere for a longer time to really take on a journey together.*" (I-GP1) |
| Recognizing opportunities | Questionnaire- What makes it difficult for you to apply MI in daily practice? “*To recognize the opportunity in time.*” (Q-GP167) |
| Teamwork | *"It [MI] is not actively supported in our General Practice (...). We have a slightly older GP, who is still a bit old-fashioned...regarding asthma/ COPD care, he prescribes medicines very often.*" (I-PN6)  Questionnaire-What else would help you to apply MI in daily practice? “*To give and receive feedback from colleagues on a regular basis. To exchange experiences.*” (Q-PN9) |
| **2. GP/PN factors** |  |
| Introduction to MI | “*Yes, you are bombarded with it. [laughs] It is a popular topic. You hear about it in different courses. You hear it from colleagues who have good experiences with it or they say: ‘I’ve gained extra time because of it [MI]’, or, ‘I get stuck with that and that patient, what should I do?’ and then it often comes down to MI*.” (I-PN2) |
| Perception of professional responsibility | *“As a PN, it [MI] is about 80% of your work.”* (I-PN7)  “*I think it [MI] is important, but it has to suit you* (…) *If it doesn’t appeal to you, you shouldn’t do it, because then it doesn’t work. (…) As a GP you have the option to refer people to the PN (…). I think it is more important that all PNs can apply [MI], as they see chronically ill patients and their work is aimed at prevention.”* (I-GP7) |
| Usefulness | Questionnaire- What has helped you so far to apply MI in daily practice? - “*Just doing it, the response of patients, results that are achieved.*” (Q-PN16)  - “*It [MI] makes conversations about behavioural change easier. Working less hard, more effect.*” (Q-GP83)  *“[Even if MI] doesn’t have the effect on health as you hoped for, the other effect, the strengthening of the doctor-patient relationship, is also very valuable. [Apart from change] it also provides the patients with more self-insight. Perhaps if something happens regarding health, those steps will be made after all. That preliminary work has been done, and that the ball can still be kicked into the goal at a later time*.” (I-GP8) |
| Self-efficacy | “*The NHG [= Dutch College of GPs] guidelines provide a very clear step-by-step plan (...) Because you are always trained in such steps, you think: ‘Well, this [MI] will be similar, just follow those steps then you’ll get there’. If you do not realize that sometimes you cannot follow those steps, because they have already been skipped by the patient ... (...) If I do not recognize those phases, then I don’t know when to switch to another phase. With some patients that is so opaque ... Then I really don’t know what to do at times.*” (I-PN1)  "*If you raise the topic, then you'll have to do something with it. Therefore, it can be safer (…) not to address it at all. However, I do think that the added value you have as a GP, is in addressing behaviour change"* (I-GP3)  Questionnaire- What makes it difficult for you to apply MI in daily practice? *- “Sometimes I don’t know which questions I can ask, I think I need to have more courage.”* (Q-PN40) *- "Because at times it still comes across as 'artificial'."* (Q-PN229) *- "Due to lack of skills/ practice it costs me a lot of energy.*" (Q-GP140) |
| Ingrained habits | “*Yes, that [falling back to old routines] happens sometimes. Wanting to give too much information. Not reasoning from the patient’s perspective. That's a pitfall (...) when it's busy or if I'm tired.*” (I-GP8)  “*With the old method (…) you don’t discuss a lot of topics or it causes a lot of frustration. I don't think I'd fall back into that [old habit] quickly.*” (I-GP5) |
| **3. Patient factors** |  |
| Level of understanding | *"Well, I try it very carefully and if I notice that it does not land, that there is no room for it, or that the subject is too complicated, then I quit. Or I do not even start when I notice in the course of the conversation: well, this is not going to work. (…) If I already have a lot of difficulty exploring what someone's complaint is, I won't take the next step, because then I expect that it will be too complicated. (…) You must be able to discuss certain basic things, someone really has to understand that, otherwise progress is hindered. Someone needs to be able to evaluate their own behavior a little bit, which requires a minimal level of intelligence. It has nothing to do with education level, because someone may not be well educated and still have good self-insight. (…) I think you have to be somewhat more directive, that people will have more benefit from that.”* (I-GP8) |
| Age | "*Adolescents often want a lot, but also forget that again quickly.*" (I-PN6)  *“There are certain groups of patients where that is a bit more difficult. And I am thinking in particular of the somewhat older patient, who is not used to this approach of the doctor.”* (I-GP2) |
| Culture | "*You cannot check whether* *he [translator] gets my message across as intended or if he simplifies my creatively formulated sentence that creates a subtle but important difference.*" (I-PN2)  "*I can speak Moroccan, so that is an advantage (...). With all kinds of cultures (...) I don't get anywhere. Some patients, and I think that has to do with culture, think: you are the professional, why do you ask me that? You just have to give me advice (...) you tell me what to do.*" (I-PN8)  "*[With ingrained] food cultures (…) it is so incredibly difficult to change that [eating habits] (...) It is a bread (...) and sugar culture."* (I-PN1) |
